# Supplementary material for: Conditional chemoconnectomics (cCCTomics) as a strategy for efficient and conditional targeting of chemical transmission
Source: eLife. 2024 Apr 30;12:RP91927. doi: 10.7554/eLife.91927 (PMC11060718; doi:10.7554/eLife.91927)
Supplement: Figure 3—source data 3. [file elife-91927-fig3-data3.zip › Figure 3 source data3 part 2.zip!/Figure 3-Source data 3-part2/1060-9-CRISPResso_Report_q0_g4o36.zip!/CRISPResso_on_1060_11/9.Alleles_frequency_table_around_sgRNA_gtcctacgtctggtgggtca.pdf]

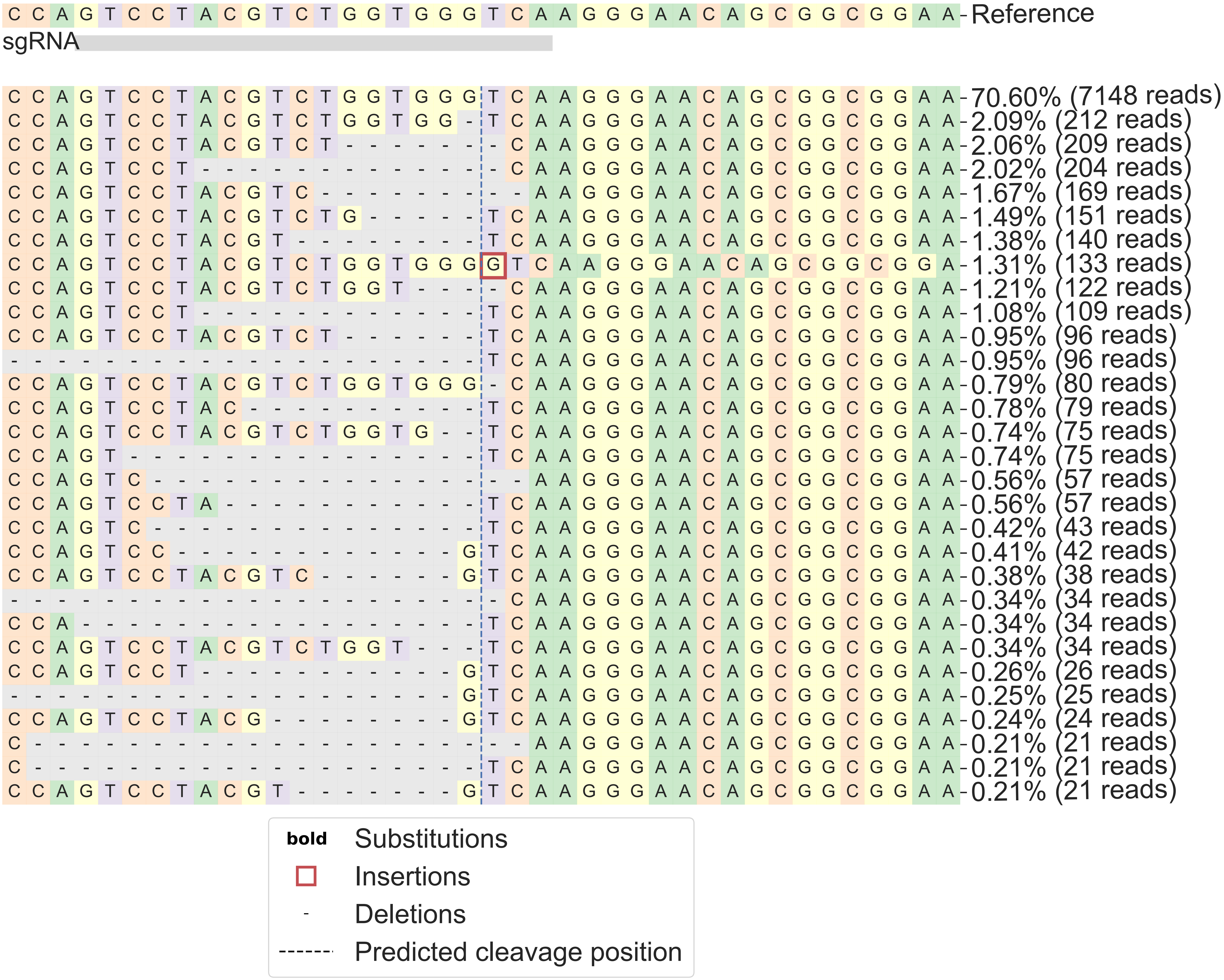

CCAGTCCTACGTCTGGTGGGTCAAGGGAACAGCGGCGGAA Reference
sgRNA
CCAGTCCTACGTCTGGTGGGTCAAGGGAACAGCGGCGGAA 70.60% (7148 reads)
CCAGTCCTACGTCTGGTGG-TCAAGGGAACAGCGGCGGAA 2.09% (212 reads)
CCAGTCCTACGTCT-------CAAGGGAACAGCGGCGGAA 2.06% (209 reads)
CCAGTCCT-------------CAAGGGAACAGCGGCGGAA 2.02% (204 reads)
CCAGTCCTACGTC---------AAGGGAACAGCGGCGGAA 1.67% (169 reads)
CCAGTCCTACGTCTG-----TCAAGGGAACAGCGGCGGAA 1.49% (151 reads)
CCAGTCCTACGT--------TCAAGGGAACAGCGGCGGAA 1.38% (140 reads)
CCAGTCCTACGTCTGGTGGGGTCAAGGGAACAGCGGCGGAA 1.31% (133 reads)
CCAGTCCTACGTCTGGT----CAAGGGAACAGCGGCGGAA 1.21% (122 reads)
CCAGTCCT------------TCAAGGGAACAGCGGCGGAA 1.08% (109 reads)
CCAGTCCTACGTCT------TCAAGGGAACAGCGGCGGAA 0.95% (96 reads)
--------------------TCAAGGGAACAGCGGCGGAA 0.95% (96 reads)
CCAGTCCTACGTCTGGTGGG-CAAGGGAACAGCGGCGGAA 0.79% (80 reads)
CCAGTCCTAC----------TCAAGGGAACAGCGGCGGAA 0.78% (79 reads)
CCAGTCCTACGTCTGGTG--TCAAGGGAACAGCGGCGGAA 0.74% (75 reads)
CCAGT---------------TCAAGGGAACAGCGGCGGAA 0.74% (75 reads)
CCAGTC----------------AAGGGAACAGCGGCGGAA 0.56% (57 reads)
CCAGTCCTA-----------TCAAGGGAACAGCGGCGGAA 0.56% (57 reads)
CCAGTC--------------TCAAGGGAACAGCGGCGGAA 0.42% (43 reads)
CCAGTCC------------GTCAAGGGAACAGCGGCGGAA 0.41% (42 reads)
CCAGTCCTACGTC------GTCAAGGGAACAGCGGCGGAA 0.38% (38 reads)
---------------------CAAGGGAACAGCGGCGGAA 0.34% (34 reads)
CCA-----------------TCAAGGGAACAGCGGCGGAA 0.34% (34 reads)
CCAGTCCTACGTCTGGT---TCAAGGGAACAGCGGCGGAA 0.34% (34 reads)
CCAGTCCT-----------GTCAAGGGAACAGCGGCGGAA 0.26% (26 reads)
-------------------GTCAAGGGAACAGCGGCGGAA 0.25% (25 reads)
CCAGTCCTACG--------GTCAAGGGAACAGCGGCGGAA 0.24% (24 reads)
C---------------------AAGGGAACAGCGGCGGAA 0.21% (21 reads)
C-------------------TCAAGGGAACAGCGGCGGAA 0.21% (21 reads)
CCAGTCCTACGT-------GTCAAGGGAACAGCGGCGGAA 0.21% (21 reads)
bold Substitutions
Insertions
- Deletions
Predicted cleavage position
